# Approaches to Modelling the Human Immune Response in Transition of Candidates from Research to Development

**DOI:** 10.1155/2014/395302

**Published:** 2014-05-07

**Authors:** Diane Williamson

**Affiliations:** Defence Science and Technology Laboratory (DSTL), Porton Down, Salisbury, Wilts SP4 0JQ, UK

## Abstract

This review considers the steps required to evaluate a candidate biodefense vaccine or therapy as it emerges from the research phase, in order to transition it to development. The options for preclinical modelling of efficacy are considered in the context of the FDA's Animal Rule.

## 1. Introduction

The development of any product for ultimate clinical use is a lengthy process, requiring the progression from proof-of-principle research into nonclinical models and safety testing and progressive phases of testing in humans, through clinical trials. The different phases of clinical trial are generally designed to progressively test the safety and immunogenicity of the candidate vaccine or therapy, usually starting with a dose-escalating design followed by dose and schedule optimisation, before pivotal trials at the selected dose and schedule.

Ordinarily, where a vaccine or therapy is directed against a disease of public health relevance which occurs predictably and regularly in a percentage of the healthy adult or pediatric population, efficacy trials of the candidate can progress in this population under authorized protocols, as long as there is sufficient nonclinical evidence that it is likely to confer benefit to this susceptible population. The endpoint of such trials is a quantifiable impact on the occurrence of, or recovery from, the disease.

However, where the vaccine or therapy is directed against a disease which is not normally prevalent, but which erupts from time-to-time in regions of the world where the disease is endemic, clinical trialling of efficacy is much more difficult. This is due not only to the unpredictability of the eruption but also the unknown size of the affected population. Furthermore, it is not ethical to deliberately expose a healthy population in a nonendemic area to potentially life-threatening disease, in order to test the efficacy of a candidate vaccine or therapy.

This situation is true for the clinical testing of vaccines or therapies directed against potential bioterrorist agents, or against pathogens that lack adequate diagnostics, or new products where a vaccine is already available but the use of a placebo arm would be unethical. All of these situations present circumstances which are both ethically challenging and which make carrying out a clinical trial of efficacy with sufficient statistical power very difficult to achieve.

In such circumstances, where clinical efficacy trials are not feasible for reasons of either logistics and/or ethics, the approval of novel vaccines for clinical use will rely on the demonstration of immune correlates of protection and the approval of novel therapies on predetermined immune readout or other endpoints [1]. This entails a clinical trial design where the endpoints are the measurement of surrogate markers of efficacy, based on immunological readouts which have been found to correlate statistically with protective efficacy in appropriate animal models. Depending on how closely the animal model mimics the human infection, more than one animal model of the infection may be required to provide immune correlates, with the following assumptions:

1. there is a well-understood pathophysiological mechanism of the toxicity of the pathogen and its prevention or substantial reduction by the vaccine or inhibition by the therapy;
2. the effect is demonstrated in one or more animal species expected to react with a response predictive for humans, unless the effect is demonstrated in a single animal species that represents a sufficiently well-characterized animal model for predicting the response in humans;
3. the animal study endpoint is clearly related to the desired benefit in humans, generally the enhancement of survival or prevention of major morbidity;
4. The data or information on the kinetics and pharmacodynamics of the vaccine or therapy or other relevant data or information, in animals and humans, allows selection of an effective dose in humans.

These concepts are embodied in the Animal Rule by the Food and Drug Agency in the USA and are discussed in detail elsewhere [1]. In a subsequent section, we will focus on how immune correlates of protection can be derived in nonclinical models and how they can be applied to predict the protection likely to be achieved in human subjects during clinical trials.

This is not to say that there are not circumstances under which non-life-threatening infections can be deliberately caused in human volunteers with their fully informed consent, in order to test a new vaccine or therapy. Indeed many such studies are authorized by ethical review bodies every year in the UK and elsewhere to test prophylaxes or therapies for influenza [2, 3]. Additionally human volunteers have given informed consent to test prophylaxes and therapies for malaria and other non-life-threatening conditions [4].

## 2. Approaches to Modelling the Human Immune Response to Candidate Vaccines or Therapies

In transitioning vaccines and therapies to the clinic, it is essential to model the human immune response as closely as possible. This is particularly important in the case of vaccines and therapies intended for use to protect against biodefense agents, since for these, it will be neither ethical nor feasible to conduct conventional phase III efficacy trials in human volunteers. Thus, for these biodefense agents it will be important to establish safety and efficacy in appropriate animal models prior to transition to Phase I safety studies [5].

In the R&D of vaccines and therapies for biodefense, the traditional approach has involved a progression from early evaluation in tissue and organ culture through an appropriate small animal model(s) to higher animal models, with the latter being engaged in the early to advanced development phases. This approach also supports a 3R's philosophy, that is, the reduction, refinement, and replacement of the use of whole animals in research, with appropriate alternatives, a policy promoted in the UK by the Home Office [6] and in the USA by the Animal Welfare Act [7] and by the Association for assessment and accreditation of laboratory animal care international (AAALAC) [8]. Tissue culture systems provide an early evaluation of candidate vaccines and therapies for their potential cytotoxic effects* in vitro* or* ex vivo* and may be of greatest value if both animal and human cell lines are available for comparison. Additionally, the ability to grow cells in a three-dimensional structure, for example, in a rotating vessel, may provide a more authentic model of the tissue or organ being simulated [9]. As a step beyond tissue culture, it is possible to maintain individual organs in a physiological medium in order to interrogate their responses to a therapeutic candidate and this has been successful, for example, with isolated, perfused lungs [10]. Furthermore,* in silico* modelling to predict what might be antigenic in microorganisms may help to make large microbial genomes more tractable and focus efforts prior to embarking on animal models [11]. There are an increasing number of algorithms available to analyse structure-function relationships, to determine surface-exposed, hydrophilic chemical groups in order to predict surface-exposed conformational or buried linear epitopes [12]. Beyond organogenic tissue culture, simple* in vivo* models such as the waxmoth larva (*Galleria mellonella*) or the nematode worm* Caenorhabditis elegans* may be used for an initial evaluation of potential toxicity and an early indication of efficacy against an administered challenge [13]. For example, the Galleria model has been demonstrated to be susceptible to challenge with bacteria of the* Burkholderia* species and can be used to evaluate approaches to therapy of these infections. Although a simple structure, Galleria provides an attractive holistic* in vivo* model in which to screen for efficacy and which does not require any of the supporting infrastructure needed to house laboratory rodents, for example [14]. Similarly,* C. elegans* has a primitive physiology and immune system and has been used to study mechanisms of infectivity and virulence used, for example, by* Pseudomonas aeruginosa* and* Staphylococcal aureus* [15]. In recent years, the zebrafish (*Dario rerio*) has gained ground as an alternative to mammalian models of infectious disease and since the sequencing of its genome, several laboratories have developed bacterial and viral disease models using the zebrafish [16]. Zebrafish have been reported to be a useful model for streptococcal infection being susceptible to the human pathogen* Streptococcus pyogenes* [17], whilst the pathology and immune response of zebrafish to* Franciscella tularensis* are similar in many aspects to that in mammals [18]. Zebrafish have also been mooted as a useful model of toll-like receptor (TLR) signalling in immunity and disease [19] and as a model for inflammatory disorders [20].

At the next level, laboratory rodents may be used to evaluate the safety, immunogenicity, and efficacy of candidate vaccines and therapies. Traditionally, the mouse has been the species of choice and over many years, a comprehensive repertoire of reagents has become available to assess the physiological and particularly the immune responses of mice. Inbred strains of mice have the advantage that their genotypes are defined and so relative differences in observed response may be related to genotype, if all other influences are standardised [21]. Non-laboratory strain, outbred mice on the other hand, may respond differently to some infections and thus may be a more authentic model for certain infections such as a Gammaherpesvirus, for which the wood mouse is a natural host [22]. Another outbred rodent model, the prairie dog, which is a zoonotic vector for* Yersinia pestis*, has recently been used to evaluate the efficacy of a candidate subunit vaccine for plague using vaccination in the wild by spiked bait [23], allowing confirmation that the vaccine is effective in both natural and laboratory models of infection [24]. Starting with a defined genotype such as that of the C57Bl6 mouse with H-2b haplotype, specific single gene deletions can be made to knock out specific cytokines or cytokine receptors or to confer sensitivity to a toxin such as diphtheria toxin [25]. The effect of these deletions may be to alter the polarity of the immune response; for example, IL10 or IL10 receptor knockouts may have a Th1-polarised response [26]; or to delete a subset of cells, for example, regulatory T-cells (Treg) in mice transgenic for diphtheria toxin (DT) receptor under the control of the* foxp3* gene locus, allowing the selective and efficient depletion of Foxp3^+^ T reg cells by the injection of diphtheria toxin [27].

Such transgenic mice still express murine major histocompatibility locus (MHC) proteins, so that their immune responses are authentically murine, hence their antigen-presenting cells (APC) will process foreign material and antigens and present peptides processed from them in the cleft of the MHCII complex on the surface of APC to murine CD4+ T-cell receptors; a T-cell response will be initiated if the T-cell receives a second signal transmitted between a B7 molecule on the APC surface and the CD28 receptor on the T-cell surface. Alternatively, processing and presentation of foreign proteins as peptides in the cleft of MHC1 molecules results in presentation to CD8+ T-cells and if a second signal is also received the CD8+ T-cell is induced to become cytotoxic. In man, the analogous system to the murine MHC system is the human leucocyte antigen (HLA) complex. HLA molecules A, B, and C correspond to MHC class 1 molecules, whereas HLA molecules DR, DQ, and DP are analogous to MHCII molecules. To exploit murine models fully to predict human immune responses, HLA transgenic mice have been developed which carry full-length genomic constructs for HLA-DR or DQ molecules and which have been crossed for many generations with C57BL/6 Ab-null mice, so that they lack expression of endogenous mouse MHC class II molecules [28]. These HLA transgenic mice thus allow an evaluation of human immune responses in a murine framework. This approach to immunoanalysis is of particular value, for example, in T-cell epitope-mapping studies where the objective is to determine the immunodominant regions of an immunogen and to assign function to structure.

Other rodent species used in the laboratory include rats, guinea pigs, and hamsters. These generally are used less frequently than mice but may be selected based on a greater resistance to microbial challenge, as with the Fischer rat [29] or on susceptibility to aerosol challenge (as with the guinea pig) [30] or as a second model of, for example,* Burkholderia* infection (hamster) [31]. Rabbits, which are classed as lagomorphs, may provide an intermediate model between rodents and non-human primates. In some cases, rabbits are a superior model to the non-human primate. For instance, in the case of modelling infection with* Bacillus anthracis* (causative of anthrax), the rabbit may be the model of choice [32] since mice are supersensitive to the capsule [33] surrounding the bacterium and the routine use of non-human primates, such as macaques, is expensive and raises ethical issues [34]. Rabbits, on the other hand, respond in a dose-related manner to spore challenge and can be protected by vaccines or therapies designed to prevent the effects of anthrax toxins [35]. In this context, the utility of HLA transgenic mice to predict human sensitivity to anthrax has been demonstrated. Using an unencapsulated strain of* B. anthracis* to avoid the supersensitivity of mice, HLA transgenic mice have been shown to be differentially susceptible, so that compared with inbred strains such as the A/J [33] and C57Bl6, HLA-transgenic mice were less susceptible [36]. Awareness of such differential resistances may help to extrapolate murine data to man or aid the selection of the most appropriate animal models with which to predict the human immune response.

Non-human primates are at the maximum level of sentience for laboratory species and should be used only with the highest level of justification and where there is no alternative [6]. Amongst NHPs, however, Rhesus and cynomolgus macaques are the most frequently used members of this Old World monkey species. Although evolutionarily most akin to humans, it cannot be assumed that NHPs will always exactly represent the human response to the testing of candidate vaccines or therapies. This was illustrated recently in the lessons learned from the testing of the super-agonistic monoclonal antibody (Mab) TGN1412 for CD28. Designed as an immune downregulator to treat inflammatory conditions such as rheumatoid arthritis, leukaemia, and multiple sclerosis, this Mab was found to be safe on extensive toxicity testing in macaques, but on testing in a Phase 1 trial in healthy human volunteers it induced pronounced inflammation with a cytokine storm, requiring intensive care of the volunteers involved [37]. This was an unexpected result since the CD28 receptor is homologous between macaques and humans and the unexpected toxicity in clinical trial volunteers prompted extensive subsequent investigation [38]. After extensive analysis of cell subtypes and their activation status it was found that, unlike in man, the CD28 receptor is not expressed on central memory effector T-cells in macaques, so that the macaques were unable to respond through this cell subset with the cytokine storm that occurred in the volunteers [39]. This investigation and analysis of the cause of the cytokine storm have led to a better understanding of some potential limitations in preclinical testing and highlighted the benefit of applying such molecular immunological approaches to preclinical testing, particularly perhaps of immunotherapeutics [39].

New World monkeys are more evolutionarily distant from man than are the Old World monkeys and comprise a group including the marmoset which may be ethically and practically (because of their small size) more acceptable as a laboratory model [40]. Recently, there has been renewed interest in the common marmoset (*Callithrix jacchus*) as a model for vaccination and therapy of microbial disease [41]. This in turn has led to the identification of a range of immunoreagents suitable for the assessment of marmoset responses [42].

For some microbial diseases, the animal model may need to be selected based on a display of similar symptomatology to that seen in man. Thus, where emesis is a common symptom, the ferret may be selected for use, as the most suitable model [43]. In the same vein, rodents are not ideal models for human inhalational exposure since they are nasal obligate breathers unlike non-human primates who can breathe through the mouth as well as the nose [44].

Infrequently, species which in the wild may transmit zoonoses may be used in captivity to test candidate vaccines or therapies. For example, the black-footed ferret, as well as the prairie dog mentioned above, has been used successfully to evaluate a recombinant subunit vaccine for* Yersinia pestis*, since both these species are able to transmit plague to man [24].

## 3. Surrogate Markers of Efficacy and Correlates of Protection

Surrogate markers of efficacy for an antimicrobial vaccine or therapy encompass immunological or microbiological readouts which explain, and are causally related to, protective efficacy and which provide endpoints which are surrogates for survival [45]. Usually, it would be expected that a number of surrogate markers would be required to predict that the candidate had induced an appropriate antimicrobial or protective immune response in man to a pathogen. The observation of statistically significant immune correlates of protection in an animal model can lead to the identification of the same immunological readouts in man, which will then serve as surrogate marker(s) of efficacy [46].

Thus, in clinical trials where the protective efficacy of the vaccine or therapy cannot be tested directly, surrogate markers of efficacy can be measured instead. For a candidate vaccine, these measurements may encompass some of the following: vaccine-specific antibody titre and functionality (e.g., toxin-neutralisation, viral plaque reduction, or bactericidal/bacteriostatic activity), cytokine secretion patterns, the induction of cell-mediated immunity with display of activation markers on immune effector cells, and an* ex vivo* proliferative or cytotoxic T cell response towards the vaccine antigen(s) or infected eukaryotic cells [47]. For a candidate therapy, these measurements may encompass the induction of appropriate responses to inhibit the binding of viruses or bacteria to host cells to prevent entry or invasion, leading to bacterial or viral clearance, or inhibition of an essential factor (e.g., bacterial cell wall assembly) for the survival and replication of bacteria or viruses in the human host [48]. Clearly, the deliberate exposure of the host to life-threatening infection to test the therapy is not ethical, so these parameters could be tested in human cells* ex vivo*. Some of these measurements may be defined as surrogate markers to substitute for survival as an endpoint in efficacy testing in man, as long as (1) the animal model in which they were derived authentically represents the human infection and (2) a statistically valid association with protection has been demonstrated in the animal model [1]. Under these conditions, the observed induction of such surrogate markers of efficacy in a clinical trial volunteer is predictive that the candidate vaccine has induced protective immunity.

Of course, it may not always be possible to identify true surrogate markers of efficacy, for example, where the entirety of the mechanism of protection is unknown. This could happen where only a single parameter in an animal model has been observed to correlate with protection but does not explain the entirety of the protective effect observed. Several scenarios have been suggested where putative surrogate markers of efficacy would not serve that function, for example, where a disease process has multiple causes and the intervention does not impact these directly, so that caution needs to be exercised to ensure that the risk : benefit ratio remains acceptable and the product being considered for licensure has tangible clinical benefit [49]. Having identified immune correlates of protection, there are various mathematical approaches which can be applied to extrapolate these nonclinical data to man in order to predict degrees of protection or therapeutic effect, which may be related to the scale of the immunological responses observed in the clinic [50].

## 4. Progress and Prospects for Licensure

Successful licensure of next generation biodefense vaccines and therapies will depend on the successful use of the FDA's Animal Rule [1], the satisfactory demonstration of immune correlates of protection across the animal species used as efficacy models and the subsequent identification of suitable surrogate markers of efficacy with which to monitor the responses of clinical trial volunteers. Recently, on the basis of the Animal Rule, several medical countermeasures have been licensed: levofloxacin (US FDA, April 2012) for inhalational anthrax, ciprofloxacin (US FDA, April 2013) for inhalational plague, and raxibacumab (US FDA, December 2012) for anthrax intoxication.

Other products which contribute to biodefense have also been licensed by the FDA, amongst which are a heptavalent botulinum antitoxin, licensed for the treatment of symptomatic botulism (2013); a reduced primary schedule with booster doses at 12 and 18 months has been approved for the FDA-licensed anthrax vaccine, AVA (Biothrax) (2012); an updated vaccinia vaccine (ACAM2000) for smallpox was approved in 2007; vaccinia immune globulin has been approved for the treatment of complications of vaccinia vaccine (2005); and an immunoglobulin for infant botulism (BabyBIG) was approved in 2003 [51].

Steady progress is being made in developing a stockpile of biodefense-related projects and subsequent papers in this issue deal with the impact of non-human primate models on understanding pathogenesis and the use of emerging technologies to understand the molecular basis of infection, prophylaxis and therapy and specific infectious disease syndromes. These advances together with guidance from the Animal Rule will have impact on the development to licensure in the long term. However, the pathway to the licensure of new biodefense vaccines is still long and challenging and current regulatory guidance is based on uncharted territory so far.
